# Supplementary material for: A Large-Scale Proteomics Resource of Circulating Extracellular Vesicles for Biomarker Discovery in Pancreatic Cancer
Source: medRxiv. 2023 Mar 20:2023.03.13.23287216. Preprint. [Version 1] doi: 10.1101/2023.03.13.23287216 (PMC10055460; doi:10.1101/2023.03.13.23287216)
Supplement: 1 [file NIHPP2023.03.13.23287216v1-supplement-1.pdf]

medRxiv preprint doi: https://doi.org/10.1101/2023.03.13.23287216; this version posted March 20, 2023. The copyright holder for this preprint (which was not certified by peer review) is the author/funder, who has granted medRxiv a license to display the preprint in perpetuity.
It is made available under a CC-BY 4.0 International license .

SUPPLEMENTARY MATERIAL

Supplementary Table 1. Baseline characteristics of patients enrolled on the discovery cohort.

Supplementary Table 2. Plasma EV analysis reproducibility

Supplementary Table 3. LC-MS results of EV analysis of plasma from patients with PDAC (PA), IPMN, Chronic Pancreatitis (CP) and Control individuals.

Supplementary Table 4. List of EV proteins that met the eligibility criteria for principal component analysis.

medRxiv preprint doi: https://doi.org/10.1101/2023.03.13.23287216; this version posted March 20, 2023. The copyright holder for this preprint (which was not certified by peer review) is the author/funder, who has granted medRxiv a license to display the preprint in perpetuity.
It is made available under a CC-BY 4.0 International license .

Supplementary Table 5. List of 182 proteins differentially expressed in PDAC compared to benign diseases.

Supplementary Table 6. List of EV proteins that are significantly altered in patients with metastatic versus non-metastatic diseases.

Supplementary Table 7. Support Vector Machine Prediction of 16 individual genes in Discovery Test and Validation Cohorts

Supplementary Table 8. List of 25 cEV proteins that met the eligibility criteria for validation studies.

Supplementary Table 9. Baseline characteristics of patients enrolled in the validation cohort.

Supplementary Figure 1. EVtrap isolation of extracellular vesicles

(A) Transmission electron microscopy (TEM) images collected of a single EV and multiple EVs captured from plasma by EVtrap. TEM imaging of EVs was carried out on a HITACHI H-8100 electron microscope (Hitachi, Tokyo, Japan) with an accelerating applied potential of 200 kV.

(B) Nanoparticle tracking analysis (NTA) of EVs after elution off EVtrap beads. NTA was carried out using ZetaView instrument (Particle Metrix) after calibration with 100 nm polystyrene particles.

medRxiv preprint doi: https://doi.org/10.1101/2023.03.13.23287216; this version posted March 20, 2023. The copyright holder for this preprint (which was not certified by peer review) is the author/funder, who has granted medRxiv a license to display the preprint in perpetuity.
It is made available under a CC-BY 4.0 International license .

Supplementary Figure 2. EV proteomics analytical performance

(A) Reproducibility of the method. A test plasma sample was processed in six replicates and performed a Pearson correlation analysis that revealed a very high correlation between replicates.

(B) Number of quantified EV proteins per sample according to different patient cohort.

Supplementary Figure 3. **Network Analyses of cEV Proteins Differentially Expressed in PDAC Compared to Benign Pancreatic Diseases.**

(A) Functional association of proteins identified by STRING database. Red, cEV proteins enriched in PDAC patients as compared to benign pancreatic diseases. Green, cEV proteins decreased in patients with PDAC as compared to benign pancreatic diseases. Red, cEV proteins increased in PDAC as compared to benign pancreatic diseases. Thickness of lines indicate confidence of association.

(B, C) Clustering of cEV protein pathways enriched **(B)** or downregulated **(C)** in PDAC cohorts. Pathways were identified using Gene Ontology database and REACTOME database.

Supplementary Figure 4. Summary of selection process to develop EV signature for pancreatic cancer diagnosis.

Supplementary Figure 5. Validation of individual cEV proteins in an independent cohort of patients.

medRxiv preprint doi: https://doi.org/10.1101/2023.03.13.23287216; this version posted March 20, 2023. The copyright holder for this preprint (which was not certified by peer review) is the author/funder, who has granted medRxiv a license to display the preprint in perpetuity.
It is made available under a CC-BY 4.0 International license .

Expression of biomarker candidates detected by Parallel Reaction Monitoring (PRM) analyses. A total of 25 cEV proteins with significant overexpression in PDAC in the discovery cohorts were quantified by PRM in a separate validation cohort of patients.

medRxiv preprint doi: https://doi.org/10.1101/2023.03.13.23287216; this version posted March 20, 2023. The copyright holder for this preprint (which was not certified by peer review) is the author/funder, who has granted medRxiv a license to display the preprint in perpetuity.
It is made available under a CC-BY 4.0 International license .

A

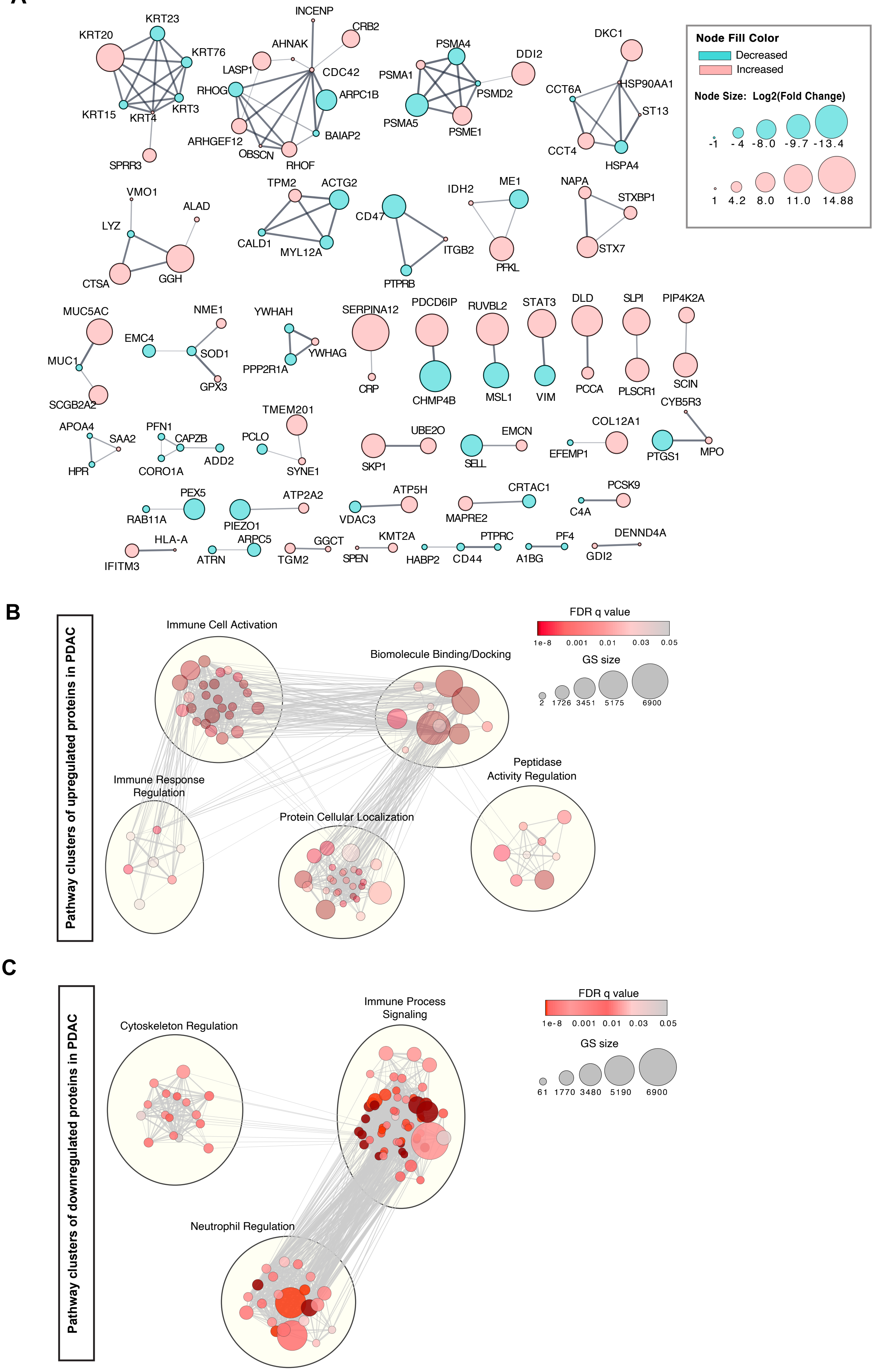

Suppl. Figure 3
